# Process evaluation of a randomised pilot trial of home-based rehabilitation compared to usual care in patients with heart failure with preserved ejection fraction and their caregiver’s

**DOI:** 10.1186/s40814-020-00747-2

**Published:** 2021-01-06

**Authors:** Karen Smith, Chim Lang, Jennifer Wingham, Julia Frost, Colin Greaves, Charles Abraham, Fiona C. Warren, Joanne Coyle, Kate Jolly, Jackie Miles, Kevin Paul, Patrick J. Doherty, Russell Davies, Hasnain Dalal, Rod S. Taylor, on behalf of the REACH-HF research group

**Affiliations:** 1 School of Nursing and Health Sciences, University of Dundee & NHS Tayside, Dundee, UK; 2 School of Medicine, University of Dundee & NHS Tayside, Dundee, UK; 3 Institute of Health Research, University of Exeter College of Medicine, Exeter, UK; 4 School of Sport, Exercise and Rehabilitation Sciences, University of Birmingham, Edgbaston, UK; 5 School of Psychological Sciences, University of Melbourne, Melbourne, Victoria 3010 Australia; 6 Institute of Applied Health Research, University of Birmingham, Birmingham, UK; 7 Research and Development, Aneurin Bevan University Health Board, St Woolos Hospital, Newport, UK; 8 REACH-HF Patient and Public Involvement Group, c/o Research development and Innovation Royal Cornwall Hospitals NHS Trust, Truro, UK; 9 Department of Health Sciences, University of York, York, UK; 10 Cardiology Department, Sandwell & West Birmingham Hospitals NHS Trust, Birmingham, UK; 11 Research, Development & Innovation, Royal Cornwall Hospitals NHS Trust, Truro, UK; 12 Primary Care, University of Exeter Medical School, Truro Campus, Truro, UK; 13 MRC/CSO Social and Public Health Sciences Unit & Robertson Centre for Biostatistics, Institute of Health and Well Being, University of Glasgow, Top floor, 200, Renfield Street, Glasgow, G2 3AX Scotland, UK

**Keywords:** Cardiac rehabilitation, Heart failure, Preserved ejection fraction, Home-based, Process evaluation, Caregivers

## Abstract

**Background:**

Whilst almost 50% of heart failure (HF) patients have preserved ejection fraction (HFpEF), evidence-based treatment options for this patient group remain limited. However, there is growing evidence of the potential value of exercise-based cardiac rehabilitation. This study reports the process evaluation of the Rehabilitation Enablement in Chronic Heart Failure (REACH-HF) intervention for HFpEF patients and their caregivers conducted as part of the REACH-HFpEF pilot trial.

**Methods:**

Process evaluation sub-study parallels to a single-centre (Tayside, Scotland) randomised controlled pilot trial with qualitative assessment of both intervention fidelity delivery and HFpEF patients’ and caregivers’ experiences. The REACH-HF intervention consisted of self-help manual for patients and caregivers, facilitated over 12 weeks by trained healthcare professionals. Interviews were conducted following completion of intervention in a purposeful sample of 15 HFpEF patients and seven caregivers.

**Results:**

Qualitative information from the facilitator interactions and interviews identified three key themes for patients and caregivers: (1) understanding their condition, (2) emotional consequences of HF, and (3) responses to the REACH-HF intervention. Fidelity analysis found the interventions to be delivered adequately with scope for improvement in caregiver engagement. The differing professional backgrounds of REACH-HF facilitators in this study demonstrate the possibility of delivery of the intervention by healthcare staff with expertise in HF, cardiac rehabilitation, or both.

**Conclusions:**

The REACH-HF home-based facilitated intervention for HFpEF appears to be a feasible and a well-accepted model for the delivery of rehabilitation, with the potential to address key unmet needs of patients and their caregivers who are often excluded from HF and current cardiac rehabilitation programmes. Results of this study will inform a recently funded full multicentre randomised clinical trial.

**Trial registration:**

ISRCTN78539530 (date of registration 7 July 2015).

**Supplementary Information:**

The online version contains supplementary material available at 10.1186/s40814-020-00747-2.

## Key messages regarding feasibility

- What uncertainties existed regarding the feasibility?

People with heart failure with preserved ejection fraction (HFpEF) have a high unmet need, experiencing low levels of health-related quality of life and an absence of evidence-based treatment options. Trials of clinical and cost-effective therapies in the HFpEF population are much needed. Rehabilitation Enablement in Chronic Heart Failure (REACH-HF) is a healthcare professional-facilitated home-based rehabilitation intervention designed to improve self-care and health-related quality of life in people with heart failure. Using qualitative research methods, this pilot trial process evaluation sought to address the uncertainties of (1) whether the REACH-HF intervention could be delivered with acceptable fidelity and (2) HFpEF patients’ and caregivers’ experiences of participation in the intervention

- What are the key feasibility findings?

Results of this process evaluation sub-study of a single-centre pilot trial showed the REACH-HF intervention was largely successfully delivered and well received by participants. However, whilst the fidelity analysis found the interventions to be delivered adequately over many of its components, we also found scope for improvement—particularly in relation to caregiver engagement.

- What are the implications of the feasibility findings for the design of the main study?

This study highlights the need for support for HFpEF patients and their caregivers. Results will guide the research team in the design and delivery of a recently funded multicentre trial of REACH-HF in this population, i.e. (1) emphasis in the patient-facing documentation used for participant recruitment of the importance of co-involvement of a caregiver (such as a spouse, family member, or friend) to actively support the patient with their engagement in the intervention; (2) enhance the facilitator training of healthcare professions to highlight both the challenges/opportunities of engaging caregivers and the key role that caregivers can bring as agents of sustainable patient behaviour change; and (3) assess the fidelity of intervention delivery to check caregiver engagement is achieved and to explore how fidelity impacts on HFpEF patient and caregiver outcomes.

## Background

In the United Kingdom (UK), approximately one million people live with heart failure (HF)—a condition which negatively affects cardiovascular functioning, often presenting with debilitating symptoms of fatigue, shortness of breath, reduced exercise capacity, and a potentially dangerous accumulation of fluid in bodily tissues [1]. Almost 50% of HF patients have preserved ejection fraction (HFpEF), and its prevalence is predicted to grow [1–3]. Although these patients are more often women, generally older, with a higher prevalence of co-morbidities (hypertension, diabetes, and atrial fibrillation) and are less likely to have coronary artery disease than those with HF with reduced ejection fraction (HFrEF), their prognosis, associated morbidity, mortality, health-related quality of life (HRQoL), and healthcare costs are comparable [1–3].

The health burden of HFpEF on patients, caregivers, the health system, and the broader economy is substantial—with markedly reduced ability to perform activities of daily living, poor health-related quality of life, and high rates of unplanned hospitalisations and associated healthcare costs, and premature mortality [4, 5]. In contrast to HFrEF, where drug and device therapies have been demonstrated to improve life expectancy and health-related quality of life, there is an absence of evidence-based treatment options for individuals living with HFpEF [6–9].

There is a growing body of evidence that exercise-based cardiac rehabilitation (CR) can benefit people with HFpEF [10]. CR is traditionally delivered in supervised group hospital-based programmes. However, given the current suboptimal uptake of CR, there is a need for alternative models of CR delivery [11]. The Rehabilitation Enablement in Chronic Heart Failure (REACH-HF) is a home-based rehabilitation intervention, facilitated by a healthcare professional, and designed to improve self-care and health-related quality of life in people with HF and their caregivers and to improve their access to CR [12].

The REACH-HFpEF pilot trial was a single-centre study with the aim of assessing the feasibility of undertaking a multicentre randomised trial to assess the clinical effectiveness and cost-effectiveness of the REACH-HF intervention in patients with HFpEF and their caregivers [13]. The patient and caregiver outcome and cost findings of the REACH-HFpEF pilot trial have been previously reported [14]. This paper presents the process evaluation sub-study of the REACH-HFpEF pilot trial that sought to assess the fidelity of intervention delivery and patients’ and caregivers’ experiences of participation in the REACH-HF intervention.

## Methods

### Design

Details of the REACH-HFpEF single-centre (Tayside, Scotland) randomised pilot trial have been published elsewhere [13, 14]. In brief, 25 HFpEF patients and 11 caregivers were allocated to either the REACH-HF intervention plus usual care (intervention group) and 25 patients and 10 caregivers to usual care alone (control group). Participating patients were aged 18 years or older and had a diagnosis of HFpEF (i.e. left ventricular ejection fraction ≥ 45%) confirmed on echocardiography, radionuclide ventriculography, or angiography within the 6 months prior to study participation.

The process evaluation included a qualitative and quantitative assessment of both the intervention fidelity (i.e. the quality and consistency of the facilitators’ delivery of the REACH-HF intervention) and a qualitative exploration of both HFpEF patient and caregiver experiences of the REACH-HF intervention, through semi-structured interviews. The quantitative fidelity results have been previously reported [14]. Intervention group participants were sampled for maximum variation based on their age, gender, presence of a caregiver, and psychological well-being (assessed by Hospital Anxiety Depression Scale (HADS) [15]) to provide a purposive sub-sample of 15 patients. Seven of these patients with their caregivers agreed to participate in the qualitative interviews. In accordance with the pilot trial protocol [13], a sample of six patients and their caregivers were selected to participate in the intervention fidelity analysis.

### REACH-HF intervention

The REACH-HF intervention is a comprehensive 12-week practitioner-facilitated self-care support programme co-designed with HF patients, caregivers, and healthcare professionals [12]. It comprises (1) a patient ‘Heart Failure Manual’ that provides information and interactive elements which target patients’ understanding of, and adaption to, living with HF, their medications, the rationale for engaging in exercise, and how to monitor and manage HF-associated symptoms and stress. The manual content was modified for this study to reflect relevant medications, causes, and treatment of HFpEF [13]; (2) a ‘Progress Tracker’ to record, review, and monitor patient symptoms, well-being, physical activity, and other self-management behaviours; (3) a choice of an exercise training programme (chair-based and/or walking); (4) information on managing stress and anxiety (including an audio relaxation CD); and (5) a ‘Family and Friends Resource’ for caregivers that provides information to help them support patients and to manage their own health and well-being.

REACH-HF participants were supported by one of two nurse facilitators (one with experience in CR and the other in HF) who had undergone a 3-day intervention training course. Over the 12 weeks, there should typically be four to six contacts with the healthcare facilitator, i.e. an initial 60–90 min face-to-face consultation at the patient’s home, up to three further ~ 30-min home visits, and two to four telephone contacts. Facilitators sought to increase the patient and caregiver understanding of living with and self-managing their HF.

### Data collection and analysis

#### Fidelity of intervention delivery

Facilitator interactions with participants were audio recorded, and the quality of delivery was assessed by an experienced researcher/cardiac nurse (KS). A sub-sample (three out of six patients) was independently checked by a second experienced qualitative researcher (JC). Scoring was discussed and compared to facilitate consistency. Listening to the detailed facilitated interactions provided additional rich data which would not have been illuminated through patients and caregiver interviews alone.

#### Semi-structured qualitative interviews

Fifteen patients, seven with caregivers, were interviewed (by JC) after the completion of their intervention in participant’s homes or by telephone, where a visit was not possible, using a pre-defined topic guide (see e-supplement). The interview assessed (1) participants’ understanding of their condition, (2) engagement with the REACH-HF intervention in supporting their adjustment to daily living with HFpEF, and (3) the perceived benefit of the intervention, including self-care behaviours and coping skills. Participants were encouraged by the researcher and through further probing to openly express their views. Interviews were audio-recorded and transcribed verbatim. Field notes were also completed (JC) to enable reflection on the process, the interviewer’s performance, and participants responses to questions and to provide contextual information to the analysis, where relevant. Reflexive memo notes were kept to assure the transparency and trustworthiness of the analysis. Thematic analysis [16] of the transcripts (led by JC and supported by KS) included data familiarisation through repeated listening to the audio recordings and review of interview transcripts. Initial codes, which summarised the content either descriptively or interpretively, were created. Codes with common features were then grouped together into emergent themes, before finally being assigned to three interpretive overarching themes. The themes are illustrated using participant quotes. Independent analysis of a sample of three transcripts by KS reflected the initial data codes, provisional themes, and sub-themes suggested by JC. Discussion and interpretation of these findings allowed refinement of themes/subthemes through development of definitions for each, as well as consideration and exploration of additional perspectives and explanations. All participants were asked if they wanted a copy of the interview transcript to review and add comments; none requested this. Both facilitators were also interviewed about their experience of delivering the intervention (by KS) using the same process described above.

Quantitative data are summarised as mean and standard deviation (SD), unless stated otherwise.

## Results

### Study participants

**Fig. 1:**
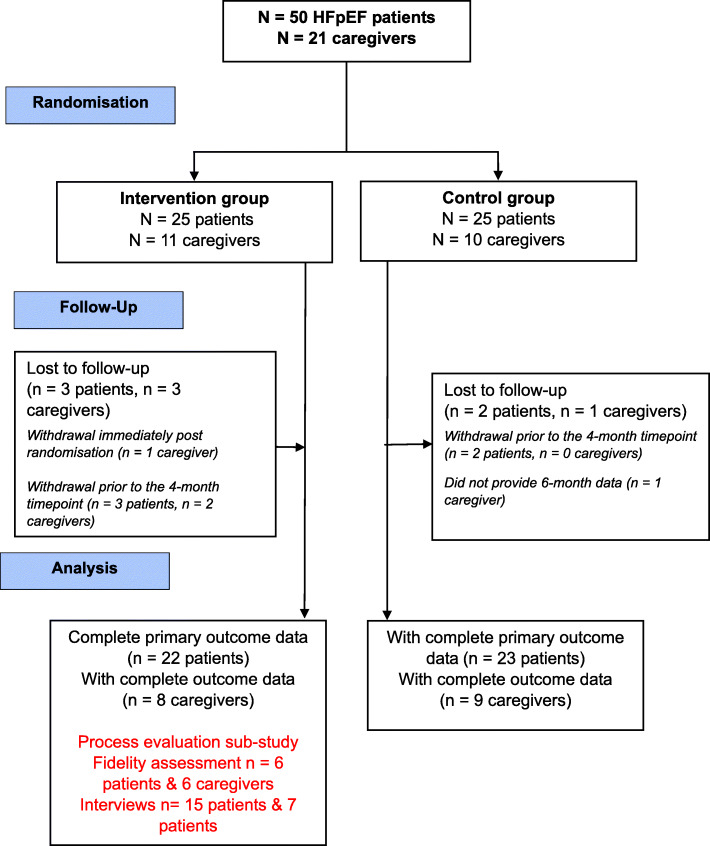
CONSORT diagram for REACH-HFpEF trial

The flow of study participants is shown in Fig. 1. Between April 2015 and June 2016, 50 patients were randomised (intervention group, *n* = 25; control group, *n* = 25). The characteristics of the 15 patients and six caregivers who participated in the process evaluation are summarised in Table 1. Patients had a mean (± SD) age of 71 (± 10.7) years with a similar proportion of men and women. Caregivers were typically a spouse or partner, younger (mean age 66 ± 10.6 years), and female. The process evaluation sample was representative of the trial intervention group [14].

**Table 1:** Baseline characteristics of HFpEF patients and their caregivers

	HFpEF patients, ***n*** = 15	Caregivers, ***n*** = 6^**a**^
Gender (female), *n* (%)	9 (60)	5 (83)
Age (years), mean (SD)	70.4 (10.6)	62.8 (10.7)
Relationship to patient, *n* (%)		
Spouse/partner		3 (50)
Sibling		2 (33)
Son/daughter		1 (17)
Number of comorbidities, *n* (%)		
0	2 (13)	
1	12 (80)	
2	1 (7)	
HADS depression, *n* (%)		
< 9	11 (73)	5 (83)
9–10	1 (7)	1 (17)
> 10	3 (20)	0 (0)
HADS anxiety, n (%)		
< 9	9 (60)	2 (33)
9–10	2 (13)	2 (33)
> 10	4 (27)	2 (33)
Living alone, n (%)	5 (33)	

^a^Data was available for 6 of the 7 caregivers

*SD* standard deviation, *HADS* Hospital Anxiety and Depression Scale

### Fidelity of intervention delivery

The six patients and caregivers included in the fidelity analysis contributed a total of 41 facilitator interactions. Of these, 34 were face-to-face contacts (mean duration 63 min, range 10 to 154 min), and seven were telephone contacts (mean duration 6 min, range 5 to 13 min). All patient/caregiver and facilitator face-to-face contacts were recorded with one exception due to audiotape malfunction. In contrast, not all telephone interactions were recorded and, in those which were, the recording quality of the patient’s conversation was poor. The content of the telephone interactions identified that they were often used to briefly ‘check in’ with participants and confirm their next scheduled face-to-face appointment, rather than assess goal setting or discuss health issues.

The audio-recording analysis of the interactions highlighted some excellent examples of the skilled facilitation using active listening skills. Facilitators listened and responded to concerns, addressed health issues, corrected misconceptions, and provided education, reassurance, and support. They facilitated goal setting and pacing within daily living and behaviour change by enabling and empowering participants to better manage their condition and engage in the REACH-HF intervention. They also instilled confidence in patients and caregivers through supportive interactions. In addition to many examples of excellent practice, this data also highlighted areas for improvement, e.g. when important cues were missed by facilitators, the absence of relatives in the interactions, and lack of caregiver interaction (even when the caregiver was physically present).

### Semi-structured qualitative interviews

All 15 patients and seven caregivers completed the interviews with a mean duration of 42 min (range 7 to 70 min), the majority undertaken either in participant’s homes (21) or one by telephone. Three overarching themes and related subthemes emerged from the analyses: (1) understanding their condition, (2) emotional consequences of HF, and (3) response to the intervention.

#### Theme 1: understanding their condition

Many participants were unaware of their HF diagnosis and its potential severity. Participants often described a protracted and uncertain path to diagnosis of their HF, with symptoms being masked by other underlying conditions and conflicting diagnostic information being provided by clinicians.

##### Reaction to diagnosis

For a few participants, particularly those who perceived themselves as ‘fit’ and healthy, their HF diagnosis was a ‘shock’ which challenged their current identity.

… I was shocked, I couldn’t believe it. I just couldn’t believe, because I’ve always been very fit [Patient interview 3]

Following diagnosis, others reported that they were ‘too frightened to do anything’. However, the majority (13 patients) did not regard their HF as fatal and believed they could accommodate it in the way they had with their other long-term conditions, seeing HF as a continuation of a biography of adaptation to illness and disability. For many patients, their diagnosis came as a relief because it normalised and explained their symptoms (e.g. tiredness and breathlessness), making them less anxious and enabling them to explain their symptoms and condition to others. The majority felt the description of HF in the REACH-HF manual, combined with the facilitator’s explanation, aided their understanding of HF and equipped them better to untangle, identify, and act on HF symptoms. Some avoided reading or asking about HF, believing this was ‘morbid’ and a source of stress which reminded them of the possibility of death.

Similar extremes in perspectives were reported by caregivers with some viewing HF as ‘final’ (a ‘death sentence’), requiring constant surveillance and the role of caregiving was extremely stressful.

I think when you get diagnosed with heart failure, from my point of view, the very word of heart failure is absolutely terrifying...And the word ‘heart failure’ is so completely final… you’re sort of…you’re never relaxing. You’re always watching to see he’s okay. You’re waking up in the middle of night, if you’ve got to get up and you’re looking at him to make sure he’s breathing… And then you think, good, he’s just sleeping…if he’s not moving, you think, why hasn’t he moved? [Caregiver Interview 20]

In contrast, others perceived that minor changes to health behaviours, such as healthy eating and exercise, were sufficient to maintain a good health-related quality of life.

#### Theme 2: emotional consequences of HFpEF

##### Loss of identity

Some participants reported restricted abilities and men especially struggled to adapt to these limitations expressing a strong sense of loss of identity. For example, one participant repeatedly expressed:

I’m not the man I used to be. [Patient interview 7]

and another that:

Everything’s been taken away. [Patient Interview 13]

They often compared their lives now to those before their illness, e.g. related to their occupational role or physical fitness. They were frustrated by how others (e.g. family, health care staff) now perceived them as individuals struggling with the constraints of their condition. Some yearned for the opportunity to demonstrate their more positive ‘former selves’, e.g. confident people with a purpose in life. One participant even expressed it may be better for himself and his family if he were dead:

I don’t want to be here, and everybody says: That’s not fair to your wife or your kids. Wait a minute, I say, Really? This is unfair to my wife and my kids. My wife deserves to be taken away for the weekend. I can’t do that. [Patient interview 13]

Caregivers confirmed this loss of social and professional roles in HF patients and acknowledged their personal challenges in managing such strong negative emotion. Caregivers highlighted the importance of regaining ‘a sense of purpose’.

… because I think he feels worthless. Sometimes I think he wishes he wasn’t here. [Caregiver interview 13b]

Instead, when patients felt useful (e.g. helping other people) or socialised and interacted with others, it lifted them emotionally and motivated them to care for themselves.

##### Recognising and responding to emotion

Patients and caregivers reported anger or low mood often related to their feelings of frustration associated with the limitations that HF imposed on their lifestyles. For six patients, the manual helped them to recognise their altered mood. Working with the facilitators enabled better management of these emotions, sometimes drawing on existing strategies, e.g. mindfulness or using new techniques, such as relaxation. Enabling patients to understand that these feelings were ‘normal’ under the circumstances allowed caregivers to support the patient’s psychological adjustment to their HF. Caregivers suggested that the intervention had reduced anxiety and improved mood, particularly in patients with elevated HADS scores. As one caregiver described:

I just feel once he started to understand more about heart failure, with the manual, that yes, he sort of - I don’t know, sort of maybe accepted it more… I think sometimes he sort of panics, thinking oh you know, should I be feeling this way? Whereas having the manual has, I think, sort of made him realise yes, this is normal for me to feel like this and be like this. [Caregiver Interview 18]

Caregivers also reported how the intervention positively addressed their own personal anxieties, thus allowing them to be more supportive. As one spouse said:

someone like myself who needs the confidence to know how to understand heart failure, to know how be less anxious... because your stress goes on to the patient … and can make them more anxious. So, if you understand maybe a little bit more about it. … you can sort of be more of a support. I think that’s what I’m trying to say. [Caregiver Interview 20]

#### Theme 3: response to REACH-HF intervention

##### Engagement with the REACH-HF intervention

While all participants engaged with the intervention at some level, this varied across the components. Participants confirmed that the REACH-HF manual provided information and reassurance: ‘offering something for everyone’. In combination with the Progress Tracker, this aided symptom monitoring and supported self-management. Patients’ and caregivers’ accounts again reinforced their need to understand how to manage HF by knowing what to look for in case of deterioration and what to do in an emergency. Through improved understanding, caregivers felt more confident in supporting the patients.

Most patients said that they followed the exercise recommendations within the manual and were able to progress satisfactorily through the chair-based exercise programme (delivered by DVD) as advised by the facilitator, either alone or together with their caregiver. A few patients needed guidance to prevent inappropriate rapid progression through the exercise levels. Facilitators proactively encouraged engagement in exercise, at times completing the chair or walking programme with resistant individuals. One participant, on the verge of giving up, described how the facilitator had supported him to identify and complete an alternative activity.

[She said] No, if you can’t do that what do you love doing? I say, I love walking.

She went, Right, if you want to go out for a walk, let’s go out for a walk.

[Patient Interview 13]

Such support provided participants with the knowledge and confidence to continue this themselves. Several caregivers who completed the chair exercise or walked with the patient valued this opportunity for social interaction and felt better emotionally afterwards. Those with a positive perception of the exercises were more strongly motivated to maintain them and integrate this into their lives. The biggest barrier to exercise was concurrent illness (e.g. chest pain) which either delayed exercise initiation or progression and episodes of acute illness (e.g. chest infection) which affected six participants and resulted in them stopping exercise for several weeks then restarting at a reduced level. Of four patients with co-ordination and balance problems, two adapted by holding the back of chairs for balance and slowly progressing through the chair exercises. In contrast, the other two patients discontinued their exercise programme.

When progress through the exercise levels did not match patients’ expectations, they were disappointed. The facilitators played an extremely important role, encouraging them, affirming progress, and suggesting more appropriate alternative exercise. Five other patients were also disheartened, feeling the chair-based exercises had not increased in intensity sufficiently nor challenged them enough even at level seven. Caregivers confirmed how the combined manual information and advice from the facilitator increased their confidence to gauge and support more appropriate levels of exercise for the patient.

The negative impact of HF on participants’ normal lifestyle and abilities could be profound as illustrated by one man who loved gardening:

I had just a little bit of turf about that long and I dug it up and whether it was the bending up or down or just pushing the shovel in that made me very unwell for I think it was about a week to recover. That’s one of the things I’ve found now that if I push myself and overdo it it’s the aftereffects that last longer [Patient interview 7]

Again, facilitators helped patients to reframe their thinking, set more realistic goals, and breakdown their activities to make them manageable through goal setting and pacing, which maintained a sense of achievement.

##### Changes in health-related behaviours

The majority (14 patients) reported some change in behaviour because of the programme. Changes included maintaining the exercise regime beyond completion of the programme (13 patients), continued symptom tracking/monitoring (eight patients), and dietary modifications (two patients). Patients who perceived immediate symptomatic benefits from the exercises were most likely to keep up their exercise regime. Others reported this had also improved their sleep patterns. Most patients claimed they knew about and followed healthy diets; two participants discussed the value of the healthy eating section. One described how involvement in REACH-HF motivated him to set new dietary goals which included healthier food shopping and more home cooking. By setting goals and applying pacing techniques, this enabled him to complete his weekly shopping which had previously been a challenge.

Only two participants continued to engage in undesirable health behaviours such as smoking and consuming a high-fat diet. They did not causally connect these behaviours to their heart disease or weight gain.

Caregivers often described facilitators as the primary motivation for behaviour change in patients, and the manual was a useful resource to complement facilitator-patient interactions. Caregivers typically encouraged and supported patients to change their lifestyles, helping them manage symptoms and engage in activities, reinforcing the facilitator’s recommendations. Some also changed their own behaviours. For example, one spouse reported:

[The facilitator] was very helpful for me in so many different ways. Helping me to understand heart failure…she encouraged me to go out walking… Just the reassurance that things were better, that there was somebody there that was willing to, erm, say, well, okay, you’re doing well. Even just the smallest amount of encouragement. And ‘my husband always felt better after [the facilitator] went away. Because she felt…almost like a little security blanket, if you want to say. That somebody was there, somebody was asking. [Caregiver Interview 20]

Feeling that someone ‘cared’, listened, answered questions, and provided feedback and encouragement was important to participants.

##### Monitoring and symptom tracking

Use of the Progress Tracker to record weekly symptom monitoring and exercise progress was variable. Some showed no desire to complete this, expressing that

Filling it all in…. that is a bit annoying you know what I mean [Patient Interview 1]

Others only completed specific sections, most commonly the weight and symptoms section. For those who found the tracker helpful, this daily symptom assessment was translated into long-term behaviour change extending beyond the REACH-HF intervention. Proactive symptom monitoring also improved patients’ abilities to communicate with doctors to allow prescribing of appropriate treatment.

In contrast, a few participants disliked the repetitiveness of the tracker, even suggesting that this at times became the focus of their interaction with the facilitator.

Every time she would come out she would start to look back through the stuff but she would go right to the front of the manual, not the manual the chart you call it, and would go through preceding weeks that she’d already covered [Patient Interview 7]

Nearly all caregivers believed that monitoring and assessing the physical and mental health of the patient was the most important but also a very difficult aspect of their role. Identifying signs and symptoms, deciding on their seriousness, and whether they related to HF or another condition then initiating appropriate action was challenging. As one spouse described:

I suppose his breathing and I know like he’s been quite concerned about sort of circulation in his legs. I don’t even know if that’s connected to the heart failure or if that’s something else, because he has got quite a few health problems. [Caregiver Interview 18]

Proximity to the patients and frequency of contact also influenced caregivers’ perceptions of their engagement in REACH-HF. One caregiver despite living over 50 miles away provided an excellent example of monitoring her relative’s physical and emotional state and adopting a ‘virtual caregivers role’ providing encouragement and emotional support through mobile technology using texts or more often FaceTime. She described the benefit of how

you can see it on him, to be honest. Sometimes he doesn’t look too good… his breathing isn’t good and he looks kind of grey… I suppose FaceTime is a different way of doing things and…. It’s lets you be involved. [Caregiver Interview 18]

Although using technology allowed her to assess his appearance, body language, and suggest interventions in a similar way to face-to-face caregivers, what differed was her limited ability to provide physical assistance. The patient could also choose not to converse over visual media (especially if they are feeling particularly unwell). This obviously undermines the virtual caregiver’s ability to assess the situation and can cause them stress, worry, and sleepless nights.

##### Unique caregiver views and experiences

Within this study, there was a strong reluctance to be identified as ‘caregivers’, even when the ‘caregiver’ assisted the patient in activities such as washing and dressing. Caregivers regarded their caring role as ‘fluid’. Most described providing minimal physical assistance on a day-to-day basis with increased help when away from home or during episodes of patient’s illness. Caregivers also highlighted how balancing competing demands on their time (e.g. caring roles for other family members), or their own health status, could affect the support they were able to give. Despite these challenges, caregivers did report examples of acting as an enabler and motivator, especially in encouraging patients to exercise, often by doing this together.

Use of the friends and family resource also varied. Some read this from cover to cover, then used it as a reference (to review the meaning of symptoms or reinforce the facilitator’s advice by referring the patient to that section of the manual) and a guide to action. However, the majority were intermittent engagers, often reading the information explaining HF or quickly glancing through it. Caregivers with no or intermittent engagement believed that the manual was primarily for the patient.

## Discussion

This process evaluation study has benefited from a qualitative approach which enabled greater understanding of the issues surrounding HFpEF and the application of REACH-HF intervention. Through multiple data sources, we observed that the intervention was largely successfully delivered and well received by participants. This study also highlights the genuine need for support in a population often excluded from many existing HF and CR services [6]. The home-based nature of the REACH-HF intervention also offers an opportunity to overcome the current challenge of suboptimal uptake of CR [1, 11].

Whilst our previously reported quantitative fidelity analysis found the REACH-HF intervention was adequately delivered by facilitators over most of its components, we also found there was scope for improvement [14]. This was particularly the case in relation to engagement with caregivers, a finding also reported with HFrEF patients [17]. Unique insights from the analysis of audio recordings of facilitator interactions provided rich data extending beyond the confines of the previously reported fidelity scores, exemplifying good practice and identifying potential areas for improvement in consultation skills. Facilitators also captured written notes of their consultations as part of a self-assessment. Complementing these with reflection on the recordings of consultation offered a powerful tool to enable self-reflection and professional development for practitioners. Caregivers believed that REACH-HF was for the patient and not for them, suggesting a more proactive strategy for caregiver involvement may be required in future intervention delivery [17].

The need to understand and know how to manage their HF reinforces earlier research in HFrEF patients [18, 19] and caregivers [20, 21]. Addressing participants’ needs for clarity in relation to their diagnosis and the implications of this condition can increase understanding, alleviate patient and caregivers’ anxiety, and allow them to accept and accommodate HF in their lives. Greater knowledge and confidence in caregivers can also enable more appropriate patient monitoring and support, confirming previously reported findings of optimised symptom management and self-care behaviour [21, 22]. Some participants valued monitoring as a measure of their progress and stability, others seeing this as a chore which has also been reported in the use of symptom monitoring diaries [23].

Increasing patient’s and caregiver’s knowledge of HF is a core element in HF care provision [24, 25]. For example, enabling them to link symptoms (e.g., breathlessness and increased body weight) allows earlier detection and prevention of HF-related deterioration. This was achieved through information provided in the manual and explanations by facilitators. The importance of feeling that someone listened and cared, acknowledged emotions, illness beliefs, anxiety, and depression, yet provided feedback and encouragement to improve self-efficacy, was all highlighted by the participants. These are critical issues in empowering patients to self-monitor and optimise their health-related quality of life [26]. Applying goal setting and pacing techniques to break down tasks (e.g. shopping, housework, and gardening) into manageable elements also allowed more proactive self-management of their condition.

The analysis from the current study suggests that HFpEF patients and their caregivers have a number of unmet needs and that the REACH-HF intervention may offer a possible solution to address this gap. The role of facilitators in implementing the REACH-HF programme is crucial. The facilitator had an important role in the prescription and support of exercise and other lifestyle change during the programme and enabled many participants to maintain exercise and dietary changes beyond completion of the programme, reflecting previous work [20]. Caregivers were often better able to recall and describe the interactions between the facilitator and the patient or themselves than the content of the REACH-HF manual. By employing counselling and coaching skills, listening to patients’ concerns, providing reassurance, reframing problems, helping them to adapt to any limitations, and motivating the patient (and the caregivers) to take exercise, the facilitator assisted both patients and caregivers in their caring role.

### Strengths and limitations

This study has a number of strengths. First, it benefited from a qualitative method approach which enabled greater understanding of the issues surrounding HFpEF. Second, REACH-HF is a home-based rehabilitation intervention for HFpEF patients (and their caregivers), a high need population with limited access to HF and rehabilitation services. Third, we successfully recruited the target number of HFpEF patients for semi-structured interviews, strengthened by including their respective caregivers, who are often excluded in HF research studies [27]. Fourth, we assessed adherence to intervention protocols and the quality of consultation interactions and explored HFpEF patients’ and caregivers’ experiences of this. Fifth, qualitative data also captured some examples of good practice in education, engagement, and support of HFpEF patients and their caregivers. Sixth, completing fidelity analysis may be a useful tool for self-reflection and improving professional practice for specialist nurses. Finally, we believe this process evaluation enhanced the reliability of the outcome results and are in keeping with the findings of the process evaluation conducted alongside our multicentre randomised controlled trial in patients with HFrEF [17]. Our learning from this study will inform future optimisation of the interventions for HFpEF [28, 29] and a full trial in a number of ways: (1) patient-facing documentation used for participant recruitment emphasising the importance of co-involvement of a caregiver (such as a spouse, family member, or friend) to actively support the patient with their engagement in the intervention; (2) enhance the training of healthcare professions facilitating the intervention and highlight both the challenges and opportunities of engaging caregivers and the key role that caregivers can bring as agents of sustainable patient behaviour change; and (3) assess the fidelity of intervention delivery to check if full caregiver engagement is achieved and to explore how it impacts on HFpEF patient and caregiver outcomes.

However, this study also had limitations. Our translation of complex interpersonal interactions into numerical scores within the fidelity analysis was unable to fully illustrate some of the excellent examples of good practice. Facilitators often demonstrated high levels of skill and competence in providing tailored educational and psychological support, enabling patients to reframe negative thoughts, engage in appropriate exercise, and participate in self-management. The sample size within this study was small, and the characteristics of the participants (predominantly of white ethnic origin) from a single centre limit the potential generalisability and may have failed to achieve theoretical saturation/information redundancy. Whilst the assessment of the fidelity of interventions by independent researchers enhanced confidence in the results, their varied professional backgrounds (nurse researcher and social scientist) may have influenced interpretations of the fidelity scoring.

## Conclusions

This process evaluation provides important evidence supporting the feasibility and acceptability of delivering the REACH-HF intervention that has the potential to address some key unmet needs in HFpEF patients and their caregivers. One of the most important intervention components identified by this study was the role of the healthcare facilitator, who acted as an educator, a source of support and reassurance, as well as a motivator and enabler. The facilitators helped to reframe participants’ thoughts to enable engagement in activity, symptom monitoring, and self-management of their HF through realistic goal setting and pacing. The study also identified how involving caregivers was at times challenging, and a more proactive strategy may be required to optimise this part of the intervention in future applications and clinical trials. The findings of this process evaluation will inform a future multicentre trial.

## Supplementary Information

**Additional file 1.** E-resource: Topic guide for qualitative interviews with patients and caregivers.

## Data Availability

Transcripts will not be shared in their entirety to protect the anonymity of participants and healthcare staff delivering the intervention. However, requests for extracts of data will be considered on reasonable individual basis from the corresponding author.

## Abbreviations

CR: Cardiac rehabilitation
HADS: Hospital Anxiety and Depression Scale
HF: Heart failure
HFpEF: Heart failure with preserved ejection fraction
HFrEF: Heart failure with reduced ejection fraction
REACH-HF: The Rehabilitation Enablement in Chronic Heart Failure
SD: Standard deviation
UK: United Kingdom
