# Supplementary material for: Process evaluation of a randomised pilot trial of home-based rehabilitation compared to usual care in patients with heart failure with preserved ejection fraction and their caregiver’s
Source: Pilot Feasibility Stud. 2021 Jan 6;7:11. doi: 10.1186/s40814-020-00747-2 (PMC7786976; doi:10.1186/s40814-020-00747-2)
Supplement: Supplementary file 1 — Additional file 1. E-resource: Topic guide for qualitative interviews with patients and caregivers. [file 40814_2020_747_MOESM1_ESM.docx]

**E-resource: Topic guide for qualitative interviews with patients and caregivers**

| **Topic** | **Questions** |
| --- | --- |
| **Opening question**  **Engagement with the intervention** | What did you know about your heart condition before you started the REACH-HF programme?  Before the programme started, what did you do to look after your heart condition?  Before you started, what were your expectations of the REACH-HF programme?  What did you want to know about?  Once the programme started, How did you use the manual? |
| **Relationship with REACH-HF facilitator** | Can you describe how the facilitator worked with you?  What did you like or dislike about how the facilitator worked with you?  What did you feel about your (husband, wife, son etc) being present?  Did the facilitator help you meet your needs? |
| **Involvement of Family and Friends** | Do you have any friends or family that help you manage your heart condition?  If so: In what way did your family or friends get involved?  How did you feel about it?  What if any discussions did you have with your family or friends about what support you needed from them in managing your heart failure? |
| **Using the manual** | As a result of using the manual and working with the facilitator what has *changed* about how you manage your heart failure?  What do you think about the medication section?  What do you think about the physical activity programme?  How do you plan to keep your fitness up in the future under your own steam? What other support might be useful?  What did you think about the progress tracker? How did you use it?  Can you tell me about what physical activity or exercise do you do now?  What are you doing to keep track of your progress? |
| **Processes** | Has taking part in the intervention affected your understanding of the condition /your situation? If so, probe further  Has it made you feel any more or less confident about how you manage your heart failure? If so, probe further |
| **Psychological adjustment to living with heart failure** | Has this programme changed the way you feel about having heart failure? If so, in what way?  Has this programme changed the way you manage these feelings? If so, in what way?  What do you think about the sections of the manual that are about managing stress? Were these helpful? If so how? How did you monitor your progress? |
| **Moderators and Mediators** | Is there anything else that affects how you look after your heart?  What do you do now to look after your heart?  What other sources of information or support do you use? This is important if there are other heart failure management programmes being used. |
| **Overall experience of the intervention**  **(quantitative question)** | Overall, how would you describe your experience of taking part in the REACH-HF programme? |
